# Left bundle branch area pacing in children: case series

**DOI:** 10.1093/ehjcr/ytaf020

**Published:** 2025-01-22

**Authors:** Ewa Jędrzejczyk-Patej, Michał Mazurek, Oskar Kowalski, Mariola Szulik, Filip Tyc, Armin Pietruczuk, Radosław Lenarczyk, Zbigniew Kalarus

**Affiliations:** Department of Cardiology, Congenital Heart Diseases and Electrotherapy, Silesian Center for Heart Diseases, Skłodowskiej-Curie 9, 41-800 Zabrze, Poland; Department of Cardiology, Congenital Heart Diseases and Electrotherapy, Silesian Center for Heart Diseases, Skłodowskiej-Curie 9, 41-800 Zabrze, Poland; Department of Human Nutrition, Faculty of Health Sciences, Medical University of Silesia, Poniatowskiego 15, Bytom, 40-055 Katowice, Poland; Department of Dietetics, Faculty of Health Sciences, Medical University of Silesia, Bytom, Katowice, Poland; Department of Cardiology, Congenital Heart Diseases and Electrotherapy, Silesian Center for Heart Diseases, Skłodowskiej-Curie 9, 41-800 Zabrze, Poland; Department of Medical and Health Sciences, WSB University Faculty of Applied Sciences, Cieplaka 1c, 41-300 Dąbrowa Górnicza, Poland; Department of Pediatric Cardiology and Congenital Heart Diseases, Faculty of Medical Sciences in Zabrze, Medical University of Silesia in Katowice, Poniatowskiego 15, 40-055 Katowice, Poland; Department of Congenital Heart Diseases and Pediatric Cardiology, Silesian Center for Heart Diseases in Zabrze, Skłodowskiej-Curie 9, 41-800 Zabrze, Poland; Division of Medical Sciences in Zabrze, Medical University of Silesia, Katowice, Poland; Department of Cardiology, Silesian Center for Heart Diseases, Skłodowskiej-Curie 9, 41-800 Zabrze, Poland; Division of Medical Sciences in Zabrze, Medical University of Silesia, Katowice, Poland; Department of Cardiology, Silesian Center for Heart Diseases, Skłodowskiej-Curie 9, 41-800 Zabrze, Poland

**Keywords:** Case series, Children, Left bundle branch area pacing, Conduction system pacing, Pacing

## Abstract

**Background:**

Left bundle branch area pacing (LBBAP) is a new concept that provides physiological pacing with a narrow QRS duration. Recently published data suggest that LBBAP may prevent deleterious effects of right ventricular pacing, namely pacemaker-induced cardiomyopathy, especially in patients with expected high ventricular pacing burden, which may be of particular importance in children.

**Case summary:**

Herein, we report successful implantation of Medtronic SelectSecure (Model 3830, Medtronic Inc.) right ventricle electrode in the region of left bundle branch area in three consecutive children (two 16-year-old and one 8-year-old). Indication for pacemaker implantation was third-degree atrioventricular block in all cases. Implantations were performed under general anaesthesia, and there were no acute complications. During the median follow-up of 6 months, there were no adverse events and the electrical parameters of the device remained stable.

**Discussion:**

Compared with adult patients, implantation of pacemakers in children may still be challenging, not only because of smaller body size but also due to continuing growth and a higher rate of lead and device-related complications. We have demonstrated that implantation of LBBAP in children is feasible and could be worth considering, particularly in patients with expected high ventricular pacing burden. Further studies are needed to assess the efficacy and safety of LBBAP in children during long-term observation.

**Learning points**

- A left bundle branch area pacing is a new concept that avoids desynchronization of the ventricular contractile and offers optimal electrical parameters.
- Our case series shows that implantation of left bundle branch area pacing in children is feasible and could be an option worth considering in patients with expected high ventricular pacing burden. During the median follow-up of 6 months, there were no adverse events and the electrical parameters of the right ventricle lead in our paediatric patients remained stable.

## Introduction

Cardiac pacing significantly improves quality of life and reduces mortality. Unfortunately, right ventricular pacing may result in pacing-induced cardiomyopathy in 15%–20% of patients who were paced for >20% of the time in a 5-year observation.^1,2^ Conduction system pacing (CSP) is a new concept that avoids the desynchronization of the ventricular contractile. Specifically, left bundle branch area pacing (LBBAP)—a modality of CSP, develops dynamically in recent years due to more optimal electrical parameters and a broader target area for lead implantation compared to His bundle pacing. The LBBAP allows for physiological pacing via a transventricular septal approach. The safety and clinical performance of LBBAP have been previously assessed.^3,4^ According to published data, the implant success rate of LBBAP in adults, in structurally normal hearts, and heart failure is ∼92% and 80%, respectively. In addition, complication rates of LBBAP and conventional stimulation are similar.^5^ The LBBAP in children has been the subject of only a few case studies. Here, we report successful implantation of LBBAP in three consecutive children and their further follow-up. All procedures were performed by two operators experienced in LBBAP implantations in adults (>100 successful procedures).

## Summary figure

**Figure:**
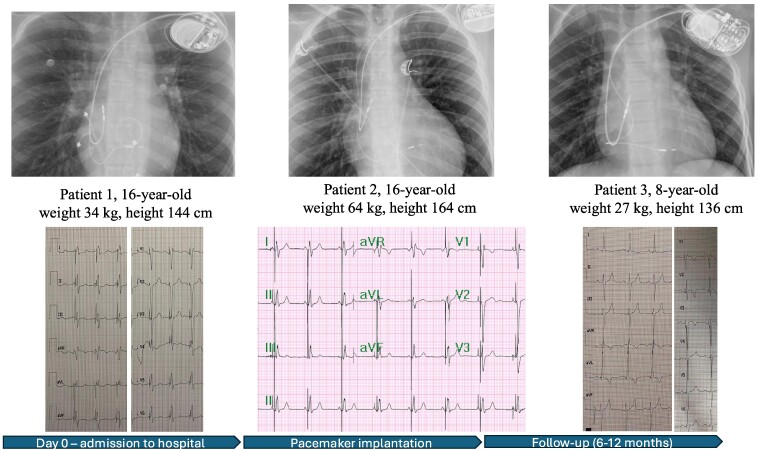


## Case presentation

### Patient 1

**Figure 1:**
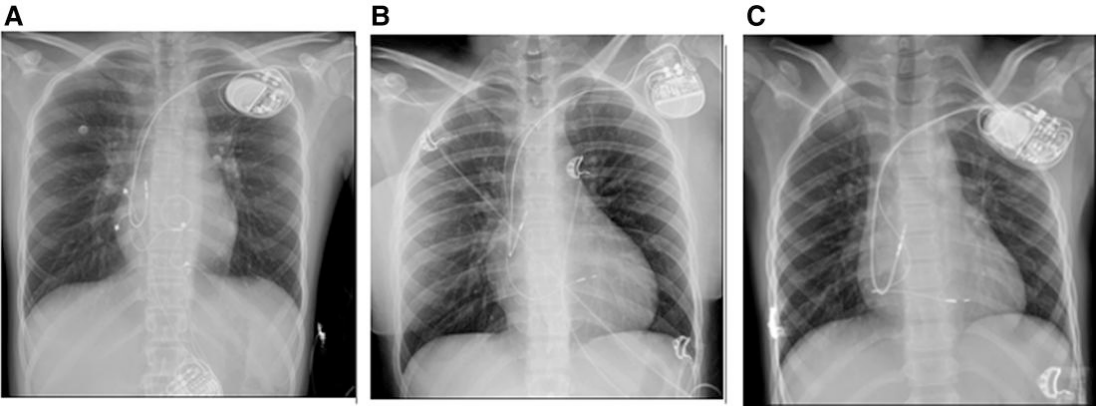
X-ray image 1 day after implantation: Case 1 (*A*), Case 2 (*B*), and Case 3 (*C*).

**Figure 2:**
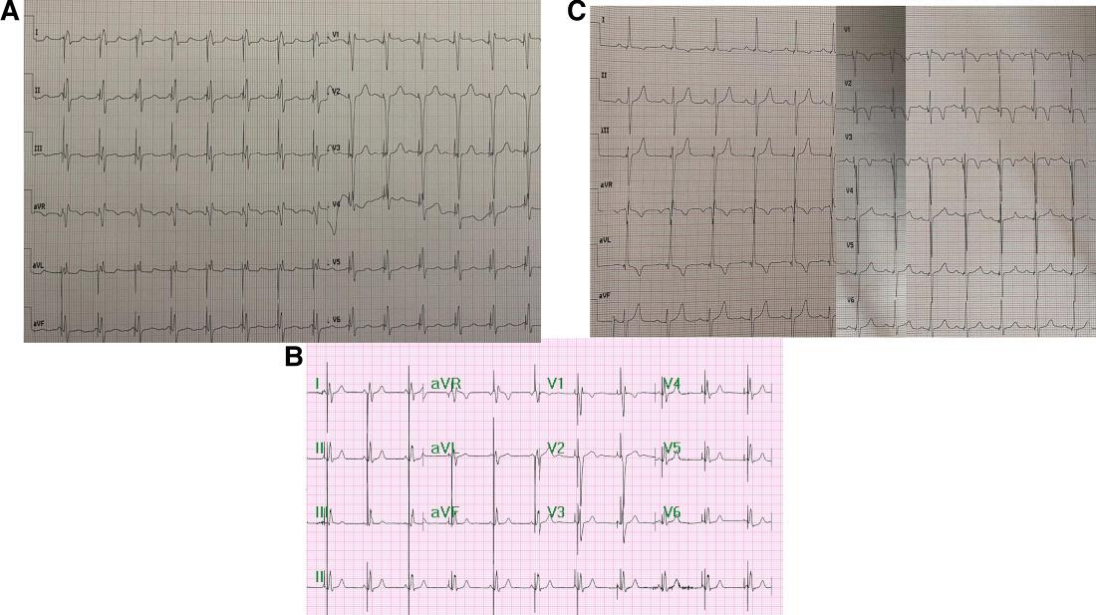
ECG 1 day after implantation: Case 1 (*A*), Case 2 (*B*), and Case 3 (*C*).

**Figure 3:**
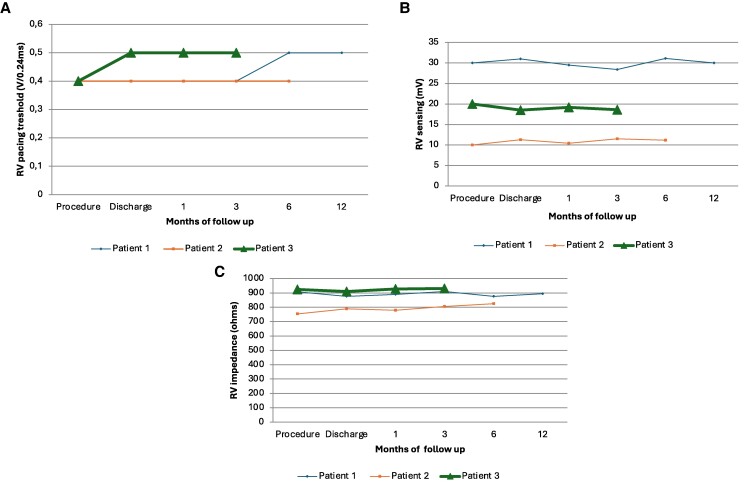
The electrical parameters of the device: right ventricle pacing threshold (*A*), right ventricle sensing (*B*), and right ventricle impedance (*C*). RV, right ventricle.

**Figure 4:**
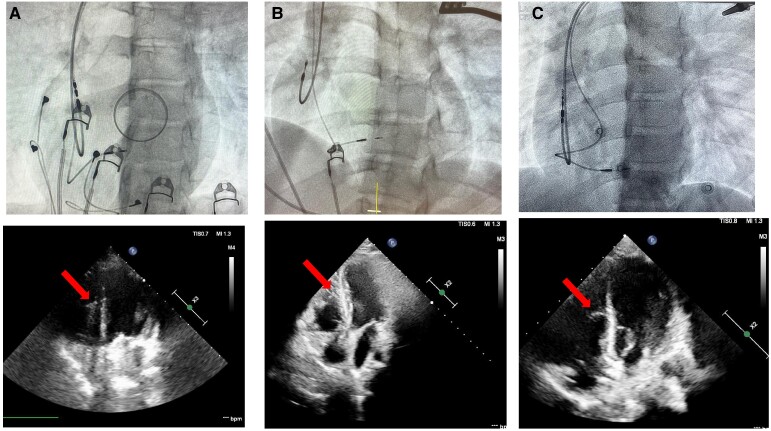
Left anterior oblique view at the end of the procedure and transthoracic echocardiography 1 day after the implantation: Case 1 (*A*), Case 2 (*B*), and Case 3 (*C*). The arrow indicates the right ventricular lead.

A 16-year-old patient, after cardiac surgical correction of a partial atrioventricular (AV) canal (ASD I), implantation of an On-X 25 mitral mechanical valve (2019), and pacemaker with epicardial leads as a result of postoperative, complete AV block (2019), was urgently admitted to the clinic due to discomfort in the region of implanted device. The patient denied heart palpitations, syncopy, or chest pain. He suffered from hypothyroidism and was underweight and undersized (body weight and height 34 kg and 144 cm, respectively). Upon admission, the patient’s general condition was good. Laboratory tests showed a low level of NT-proBNP (124 pg/mL; *N* < 125 pg/mL) and mild hypokalaemia. Transthoracic echocardiography (TTE) showed a heart with normal contractility and proportions and an artificial valve in the mitral position with a good function and a flow gradient of 14/6 mmHg. A pacemaker check revealed a sudden increase in ventricular threshold (temporally coinciding with symptoms reported by the patient), periodic lack of ventricular stimulation, and pacemaker dependency due to third-degree AV block. After consultation with an electrophysiologist, the patient was qualified to implant a transvenous dual-chamber pacemaker. A written informed consent was obtained from parents before the procedure. Implantation was performed under general anaesthesia in November 2023. A Medtronic SelectSecure (Model 3830 59 cm, Medtronic Inc.) right ventricle electrode was implanted via the left axillary vein puncture (under ultrasound guidance) into the interventricular septum (into the region of the intermediate fascicle of the left bundle branch). The sheath C315 His (Medtronic Inc.) was used for lead delivery. The R-wave peak time (RWPT) was 63 ms, and interpeak V1–V6 was 45 ms. The transition of QRS morphology while pacing with a decreasing ventricular amplitude was not observed. A myocardial current of injury (COI) increase was initially observed as the lead penetrated the septum, followed by a decrease as the lead reached the left ventricular (LV) subendocardial area. The right atrium Biotronik electrode was implanted in the right atrial appendage. Lead parameters were correct. A Biotronik Enitra 6 DR-T dual-chamber pacemaker was connected to the electrodes and placed in the subcutaneous pocket. Parameters of the right ventricle lead after the procedure are presented in *Table 1*. DDDR stimulation with a lower rate of 70/b.p.m. was programmed with an AV delay of 110 ms and unipolar ventricular stimulation. The total fluoroscopy and procedure duration were 7 min, 20 s, and 130 min, respectively, whereas the fluoroscopy dose was 7.51 mGy. There were no peri- or post-procedural complications. The location of the leads is shown in *Figure 1A*. The patient was discharged home on the fifth day post-implantation. Since then, the child has been carefully observed in the cardiology outpatient clinic. During the 12-month follow-up, there were no adverse events with the proper device function, stable sensing, threshold, and impedance parameters of RV lead, and stable morphology of QRS in ECG (*Figure 2A*). The information about the patient is summarized in *Table 1*. The electrical parameters of the device are presented in *Figure 3*, and the location of the right ventricle electrode has been shown in *Figure 4A*.

**Table 1:** Summary of cases

	Patient 1	Patient 2	Patient 3
Age (years)	16	16	8
Height (cm)	144	164	136
Weight (kg)	34	64	27
Procedure duration (min)	130	95	120
Fluoroscopy dose (mGy)	7.51	5.85	7.09
RWPT (ms)	63	69	45
Interpeak V1–V6 (ms)	45	35	17
Transition	No	Yes, in programmed stimulation	Yes, at 1.7 V/0.4 ms stimulation
Lead location of CSP	Intermediate fascicle of the left bundle branch	anterior fascicle of the left bundle branch	posterior fascicle of the left bundle branch
Intrinsic QRS duration (ms)	115	80	80
QRS duration (ms) after implantation	120	115	100
R-wave (mV)	30	10	20
RV threshold (V/0.4 ms)	0.4	0.4	0.4
Impedance (ohms)	907	754	925
Vp percentage after the procedure	100%	100%	22%

CSP, conduction system pacing; RWPT, R-wave peak time; Vp, ventricular pacing.

### Patient 2

A 16-year-old patient with an AV third-degree block that was found accidentally at the age of 2 was admitted to the hospital. The patient was appropriately developed (body weight and height of 64 kg and 164 cm, respectively) and tolerated exercise well. The TTE showed a heart without a structural defect and a slightly changed left ventricle geometry. A cardiac magnetic resonance imaging (MRI) revealed slightly enlarged left and right ventricles with a normal LV ejection fraction (LVEF 59%). Laboratory tests showed no significant abnormalities. A Holter ECG showed a third-degree AV block with a ventricular rhythm of 37–88 b.p.m. (47 b.p.m. at average). The patient was qualified for pacemaker implantation. A written informed consent was obtained from parents before the procedure. Implantation was performed under general anaesthesia in May 2024. Medtronic SelectSecure (Model 3830 59 cm, Medtronic Inc.) right ventricle electrode was implanted via left axillary vein puncture (under ultrasound guidance) in the interventricular septum (in the region of the anterior fascicle of the left bundle branch). The C315 His sheath (Medtronic Inc.) was used for lead delivery. The right atrium Boston Scientific electrode was implanted to the right atrial appendage. Lead parameters were correct. A Boston Scientific Proponent dual-chamber pacemaker was connected to the electrodes and placed in the subcutaneous pocket. Parameters of the right ventricle lead after the procedure, the total fluoroscopy, and the procedure duration are presented in *Table 1*. DDDR stimulation with a lower rate of 60/b.p.m. was programmed with AV delay of 100 ms and unipolar ventricular stimulation. The RWPT was 69 ms, interpeak V1–V6 was 35 ms, and the transition of QRS morphology was observed during programmed ventricular stimulation. There were no peri- or post-procedural complications. The location of the leads is shown in *Figure 1B*. The patient was discharged home 7 days after the implantation and has been closely monitored in the cardiology outpatient clinic. During the 6-month follow-up, there were no adverse events, and the parameters of sensing, pacing threshold, and impedance of RV lead remained stable. The QRS morphology was the same as in the post-implantation ECG (*Figure 2B*). The information about the patient is summarized in *Table 1*. The electrical parameters of the device are presented in *Figure 3*, and the location of the right ventricle electrode has been shown in *Figure 4B*.

### Patient 3

An 8-year-old patient with a history of loss of consciousness, first-degree AV block (PQ 260 ms), paroxysmal second- and third-degree block, with a pause of 4.6 s in the Holter ECG monitoring, was urgently admitted to the hospital. Upon admission, the patient was in good general condition and haemodynamically stable. Transthoracic echocardiography showed a slightly enlarged left ventricle, with a rounded heart apex and preserved global systolic function of both ventricles. Laboratory tests showed no significant abnormalities. Lyme and autoimmune diseases were ruled out, and cardiac MRI showed no significant abnormalities. The body weight and height of the patient were 27 kg and 136 cm, respectively. The patient was qualified for pacemaker implantation. Written informed consent was obtained from parents before the procedure. Implantation was performed under general anaesthesia in June 2024. Medtronic SelectSecure (Model 3830 59 cm, Medtronic Inc.) right ventricle electrode was implanted via the left axillary vein (under ultrasound guidance) in the interventricular septum (in the region of the posterior fascicle of the left bundle branch). The C315 His sheath (Medtronic Inc.) was used for the lead delivery. The Medtronic electrode was implanted into the right atrial appendage. Lead parameters were correct. A Medtronic Astra dual-chamber pacemaker was connected to the electrodes and placed in the subcutaneous pocket. Parameters of the right ventricle lead after the procedure, the total fluoroscopy, and the procedure duration are presented in *Table 1*. DDDR stimulation with a lower rate of 60 b.p.m. was programmed with AV delay of 140 ms and bipolar ventricular stimulation. The RWPT was 45 ms, interpeak V1–V6 was 17 ms, and the transition of a QRS morphology was observed at the threshold of 1.7 V/0.4 ms. There were no peri- or post-procedural complications. The location of the leads is shown in *Figure 1C*. The patient was discharged home 7 days post-implantation and has been closely monitored in the outpatient clinic ever since. At the 5-month follow-up, there were no adverse events, with stable parameters of RV lead and stable morphology of QRS in ECG (*Figures 2C* and *3C*). The information about the patient is summarized in *Table 1*, and the location af the right ventricle electrode has been shown in *Figure 4C*.

## Discussion

Our case series demonstrates that the implantation of LBBAP is feasible and safe in children, even in smaller ones (weight <30 kg), including various clinical scenarios, e.g. prior cardiothoracic surgery or the presence of mechanical heart valves. To our knowledge, only a few case series on implanting a CSP system in paediatric patients have been published previously.^6^ The largest study to date showed the safety and feasibility of His bundle pacing in 24 paediatric patients or patients with congenital heart disease,^7^ 1 case report documented selective LBBP in a 13-year-old child with congenital complete heart block,^8^ and 2 other studies reported successful LBBAP implantation in 12 and 21 children in a Chinese population^9,10^ with the average age between 3 and 14 years, and 3.3 ± 2.1 years old, respectively.

The implantation of pacemakers in children is still challenging due to their smaller body size, continuing growth, and high level of physical activity. There is also a higher rate of lead and device-related complications compared to older patients.^11,12^ Moreover, a high proportion of right ventricle pacing is a known risk factor for pacing-associated cardiomyopathy in the paediatric population.^13,14^ His bundle pacing has been described as the most physiological pacing. However, it has several significant limitations, such as higher pacing thresholds over time with sooner battery depletion, observed atrial oversensing, or potential loss of capture due to the progression of the AV block more distally during long-term observation. These hazards were not observed with LBBAP, which also provides physiological pacing with a narrow QRS duration. In our case series, the average paced QRS duration was 111 ms, only 20 ms wider than intrinsic QRS. These data are compatible with previously published cases. Paediatric patients seem to achieve shorter RWPT than adults. In our patients, RWPT was ≤69 ms, whereas in adults, RWPT ≤75 ms is sensitive and specific for LBB capture.^15^ However, optimal values of RWPT and interpeak V1–V6 in children remain to be established.

The septum in children is thinner and might be about 5–6 mm^9^; thus, LBBAP implantation could potentially be more difficult, and the risk of perforation might be higher. In our case series, we chose the lumenless electrode for implantation in children because it has a smaller diameter, is less traumatic, and might result in less risk for collateral damage to septal vessels or septal injury as compared with stylet-driven electrodes.^3^ While implanting the electrode, we were screwing the electrode very carefully, constantly observing the electrode position and progression within the septum and COI to avoid perforation. We assess proper penetration within the septum (perpendicular to the septum position of the electrode while screwing in) by continuous fluoroscopy in the left anterior oblique 30° and right anterior oblique 30°, unipolarly paced QRS morphology, fixation beats, myocardial COI, and unipolar pacing impedance. In addition, we assess anodal capture (from the ring of the electrode), which also informs us about electrode depth within the septum. Although the septum in children is thinner, we observe the same COI changes and impedance values during lead implantation as in adult patients. Also, the fixation beats are frequent. Anodal pacing from the ring of the electrode was observed in two out of three cases. We did not observe any acute perforation or within the follow-up (FU) period in any case. Fortunately, from observations in adult patients, once a perforation is noted, it requires lead reposition/replacement, usually without any complication. Based on our limited experience with LBBAP in children, we must admit, we have not observed any major differences as compared to adult patients. This applies to the procedure itself, observed and measured parameters, as well as acute and long-term performance (>1 year of FU). The major question of whether LBBAP indeed prevents pacemaker-induced cardiomyopathy requires large-scale studies with long observation.

Another open question remains about lead extraction in young patients. As demonstrated recently, CSP lead extraction is feasible and safe in adult subjects.^16^ As LBBAP is developing very dynamically, it is expected that tools for removing these electrodes will also be available in the future. Till now, only a 1.5-year follow-up of LBBAP in children has been reported. In our case series, one child was observed for 12 months and two others for 6 and 5 months, respectively. During that period, no complications were observed. Moreover, the LBBAP lead parameters remained stable with a low pacing threshold and stable sensing parameters. Of note, our experience and published data refer to Medtronic electrodes in paediatric patients only.

## Conclusion

We have demonstrated that the implantation of LBBAP in children is possible and worth considering in patients with high ventricular pacing burden. However, further studies are needed to assess the feasibility and safety of LBBAP in children in long-term observation.

## Data Availability

The data underlying this article are available in the article.

## Lead author biography

Ewa Jędrzejczyk-Patej is a cardiologist. She has graduated from the Medical University of Silesia in Katowice. She is working at the Department of Cardiology, Congenital Heart Diseases and Electrotherapy, Silesian Center for Heart Diseases in Zabrze since 2008. Her specialty is clinical electrophysiology. She is a Fellow of the European Society of Cardiology, an EHRA member, and a member of the Polish Cardiac Society and Polish Heart Rhythm Section.

## Notes

**Consent:** The authors confirm that written consent for submission and publication of this case report has been obtained from the patient in line with COPE guidance.

**Funding:** None declared.

## References

[1] Kiehl EL , Makki T, Kumar R, Gumber D, Kwon DH, Rickard JW, et al Incidence and predictors of right ventricular pacing-induced cardiomyopathy in patients with complete atrioventricular block and preserved left ventricular systolic function. Heart Rhythm 2016;13:2272–2278. 27855853 10.1016/j.hrthm.2016.09.027

[2] Khurshid S , Epstein AE, Verdino RJ, Lin D, Goldberg LR, Marchlinski FE, et al Incidence and predictors of right ventricular pacing-induced cardiomyopathy. Heart Rhythm 2014;11:1619–1625. 24893122 10.1016/j.hrthm.2014.05.040

[3] Burri H , Jastrzebski M, Cano Ó, Čurila K, de Pooter J, Huang W, et al EHRA clinical consensus statement on conduction system pacing implantation: endorsed by the Asia Pacific Heart Rhythm Society (APHRS), Canadian Heart Rhythm Society (CHRS), and Latin American Heart Rhythm Society (LAHRS). Europace 2023;25:1208–1236. 37061848 10.1093/europace/euad043 PMC10105878

[4] Jastrzębski M , Kiełbasa G, Cano O, Curila K, Heckman L, De Pooter J, et al Left bundle branch area pacing outcomes: the multicentre European MELOS study. Eur Heart J 2022;43:4161–4173. 35979843 10.1093/eurheartj/ehac445 PMC9584750

[5] Huang W , Su L, Wu S, Xu L, Xiao F, Zhou X, et al A novel pacing strategy with low and stable output: pacing the left bundle branch immediately beyond the conduction block. Can J Cardiol 2017;33:1736.e1–1736.e3. 10.1016/j.cjca.2017.09.013 29173611

[6] Chubb H , Mah D, Dubin AM, Moore J. Conduction system pacing in pediatric and congenital heart disease. Front Physiol 2023;14:1154629. 37035676 10.3389/fphys.2023.1154629 PMC10080025

[7] Gordon A , Jimenez E, Cortez D. Conduction system pacing in pediatrics and congenital heart disease, a single center series of 24 patients. Pediatr Cardiol 2024;45:1165–1171. 35678827 10.1007/s00246-022-02942-9 PMC11252089

[8] Ponnusamy SS , Muthu G, Bopanna D. Selective left bundle branch pacing for pediatric complete heart block. Indian Pacing Electrophysiol J 2020;20:78–80. 31866553 10.1016/j.ipej.2019.12.012 PMC7082648

[9] Li J , Jiang H, Zhang Y, Cui J, Li M, Zhou H, et al A study to analyse the feasibility and effectiveness of left bundle branch area pacing used in young children. Pediatr Cardiol 2024;45:681–689. 36840807 10.1007/s00246-023-03119-8

[10] Wenlong D , Baojing G, Chencheng D, Jianzeng D. Preliminary study on left bundle branch area pacing in children: clinical observation of 12 cases. J Cardiovasc Electrophysiol 2022;33:1558–1566. 35508760 10.1111/jce.15520

[11] Helguera ME , Maloney JD, Pinski SL, Woscoboinik JR, Wilkoff BL, Castle LW. Long-term performance of endocardial pacing leads. Pacing Clin Electrophysiol 1994;17:56–64. 7511232 10.1111/j.1540-8159.1994.tb01351.x

[12] Olgun H , Karagoz T, Celiker A, Ceviz N. Patient- and lead-related factors affecting lead fracture in children with transvenous permanent pacemaker. Europace 2008;10:844–847. 18448424 10.1093/europace/eun109

[13] Czosek RJ , Gao Z, Anderson JB, Knilans TK, Ollberding NJ, Spar DS. Progressive QRS duration and ventricular dysfunction in pediatric patients with chronic ventricular pacing. Pediatr Cardiol 2021;42:451–459. 33247765 10.1007/s00246-020-02504-x

[14] Gebauer RA , Tomek V, Salameh A, Marek J, Chaloupecký V, Gebauer R, et al Predictors of left ventricular remodelling and failure in right ventricular pacing in the young. Eur Heart J 2009;30:1097–1104. 19286675 10.1093/eurheartj/ehp060 PMC2675702

[15] Jastrzębski M , Kiełbasa G, Curila K, Moskal P, Bednarek A, Rajzer M, et al Physiology-based electrocardiographic criteria for left bundle branch capture. Heart Rhythm 2021;18:935–943. 33677102 10.1016/j.hrthm.2021.02.021

[16] Vijayaraman P , Trivedi RS, Koneru JN, Sharma PS, De Pooter J, Schaller RD, et al Transvenous extraction of conduction system pacing leads: an international multicenter (TECSPAM) study. Heart Rhythm 2024;21:1953–1961. 38762819 10.1016/j.hrthm.2024.04.054

